# Single-cell Omics Assessment of Mitochondrial Function: Current Status and Future Perspectives

**DOI:** 10.1093/gpbjnl/qzaf081

**Published:** 2025-09-17

**Authors:** Xu Zhang (张旭), Peng An (安鹏), Zhengyang Zhang (张正阳), Yanling Hao (郝彦玲), Xiaoxian Guo (郭晓贤), Yinhua Zhu (朱银华), Zhenglong Gu (顾正龙), Yongting Luo (罗永挺), Junjie Luo (罗俊杰)

**Affiliations:** Department of Nutrition and Health, Beijing Advanced Innovation Center for Food Nutrition and Human Health, China Agricultural University, Beijing 100193, China; Department of Nutrition and Health, Beijing Advanced Innovation Center for Food Nutrition and Human Health, China Agricultural University, Beijing 100193, China; Department of Nutrition and Health, Beijing Advanced Innovation Center for Food Nutrition and Human Health, China Agricultural University, Beijing 100193, China; Department of Nutrition and Health, Beijing Advanced Innovation Center for Food Nutrition and Human Health, China Agricultural University, Beijing 100193, China; Tianjin Institute of Industrial Biotechnology, Chinese Academy of Sciences, Tianjin 300308, China; Department of Nutrition and Health, Beijing Advanced Innovation Center for Food Nutrition and Human Health, China Agricultural University, Beijing 100193, China; Greater Bay Area Institute of Precision Medicine (Guangzhou), Fudan University, Guangzhou 511400, China; Institute of Life Sciences, Fudan University, Shanghai 200433, China; Department of Nutrition and Health, Beijing Advanced Innovation Center for Food Nutrition and Human Health, China Agricultural University, Beijing 100193, China; Department of Nutrition and Health, Beijing Advanced Innovation Center for Food Nutrition and Human Health, China Agricultural University, Beijing 100193, China

**Keywords:** Single-cell, Omics, Mitochondrion, Heterogeneity, Pathogenic mechanism

## Abstract

In recent years, significant advancements in single-cell omics technologies have offered new insights into the study of mitochondria. These technologies are particularly suitable for investigating mitochondria due to their capacity to address both intracellular and intercellular heterogeneity. In this review, we categorize the mitochondrial dysfunction and variability identified in both pathological and physiological contexts through single-cell omics assessments. We examine the cutting-edge single-cell omics technologies and trace the evolution of studies on mitochondria, highlighting the transition from low-throughput to high-throughput capabilities and from single data types to the integration of multiple genomic and phenomic profiles. Furthermore, we emphasize the applications of single-cell mitochondrial assessment methods in exploring disease mechanisms, screening, and prevention, as well as their potential impacts on lineage tracing, drug discovery, and genetic counseling. Insights gained from single-cell technologies may lead to the development of novel therapeutic strategies, offering promising avenues for addressing diseases associated with mitochondrial dysfunction. Lastly, we identify the limitations of current methodologies and propose areas of focus for future research.

## Graphical abstract

**Figure:**
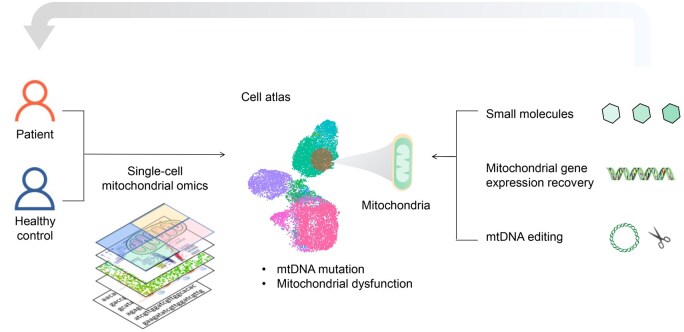

## Introduction

**Figure 1:**
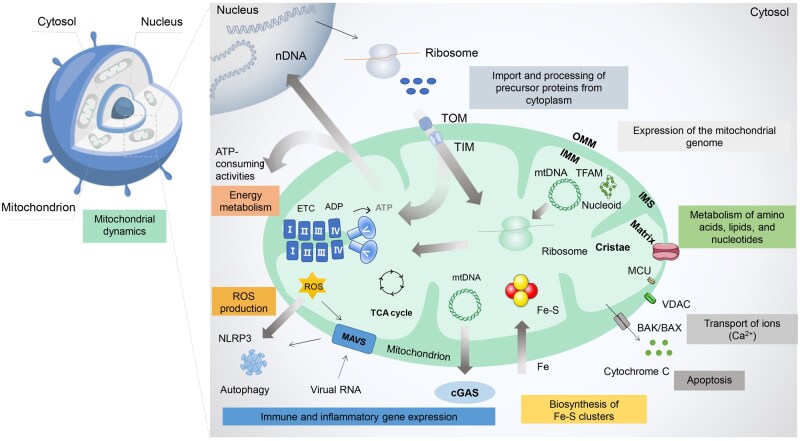
Mitochondrial structure and functions Mitochondria consist of the OMM and the IMM. The compartments divided by the two membranes are IMS and matrix. Mitochondria are responsible for a wide variety of functions. The main functions and related molecules are indicated in the figure: expression of the mitochondrial genome; metabolism of amino acids, lipids, and nucleotides; transport of ions; apoptosis; biosynthesis of Fe-S clusters; assembly of the ETC and energy metabolism; ROS production; import and processing of precursor proteins synthesized in the cytoplasm; and mitochondrial dynamics. The main elements of the proposed model are available at http://smart.servier.com/ (accessed on 7 September 2023). ADP, adenosine diphosphate; ATP, adenosine triphosphate; cGAS, cyclic GMP-AMP synthase; ETC, electron transport chain; MAVS, mitochondrial antiviral signaling protein; MCU, mitochondrial calcium uniporter; mtDNA, mitochondrial DNA; NLRP3, NOD-like receptor thermal protein domain associated protein 3; ROS, reactive oxygen species; TCA, tricarboxylic acid; TIM, translocase of the inner mitochondrial membrane; TOM, translocase of the outer mitochondrial membrane; VDAC, voltage-dependent anion-selective channel; OMM, outer mitochondrial membrane; IMM, inner mitochondrial membrane; IMS, intermembrane space; Fe-S, iron-sulfur; nDNA, nuclear DNA; TFAM, transcription factor A, mitochondrial; BAK, Bcl-2 antagonist/killer; BAX, Bcl-2-associated X protein.

Mitochondria, characterized by their distinctive double membrane structure and cristae, are essential organelles within eukaryotic cells. The dynamic processes of membrane fusion and fission allow mitochondria to interact and communicate, either forming interconnected networks or remaining isolated to effectively respond to environmental stressors [1]. These organelles are responsible for a wide array of functions related to cellular metabolism and homeostasis (**Figure 1**). Key mitochondrial functions encompass energy metabolism, which is intricately linked with cellular metabolic processes and signaling pathways. Notably, mitochondria exhibit distinct characteristics in terms of proteome composition, metabolite profiles, and substrate oxidation preferences across different cell types [2]. Additionally, mitochondria contain a circular genome, mitochondrial DNA (mtDNA), which encodes 13 proteins typically matured within mitochondria [3]. Notably, the *CYTB* gene exhibits dual functionality: it encodes both a standard mitochondrial protein and the 14th mitochondrial protein, CYTB-187AA, which is synthesized in the cytoplasm [4]. The accumulation of heteroplasmic pathogenic mtDNA mutations beyond a certain threshold can lead to deficiencies in the electron transport chain (ETC).

Since the initial description of mitochondria in the 19th century, our understanding of their structural and functional diversity, along with their roles in human health, has significantly expanded. However, due to the heterogeneity and dynamic nature of mitochondria, several challenges remain to be addressed. First, the pathogenic genes and mutations associated with numerous mitochondria-related diseases have yet to be fully identified. Results from bulk sequencing provide an averaged representation of mtDNA heterogeneity or mitochondrial gene expression levels across entire tissues, potentially obscuring harmful mutations and specific cell types within tissues that exhibit high heterogeneity, such as tumors and the placenta. Second, mitochondrial diseases present considerable challenges in diagnosis, screening, and treatment. Both the mitochondrial and nuclear genomes contribute to mitochondrial function, meaning that patients with identical mutations can exhibit varying phenotypes. In terms of screening, the genetic heterogeneity of mtDNA may result in low-level mutations being masked in the early stages when using bulk sequencing. Furthermore, the multi-copy nature of mtDNA complicates mitochondrial gene therapy compared to nuclear gene therapy. Lastly, given the diverse functions of mitochondria, assessing mitochondrial status solely based on adenosine triphosphate (ATP) production is insufficient. A comprehensive evaluation requires the integration of data from various modalities, including mitochondrial mutations, gene expression, morphology, and reactive oxygen species (ROS) levels. Fortunately, many of these issues can be effectively addressed through the application of single-cell technologies, even in their current developmental state.

In this review, we highlight the ways in which contemporary single-cell technologies enhance our understanding of mitochondria. Furthermore, we discuss future directions for single-cell mitochondrial research and explore their potential applications in promoting human health.

## Mitochondrial heterogeneity

**Figure 2:**
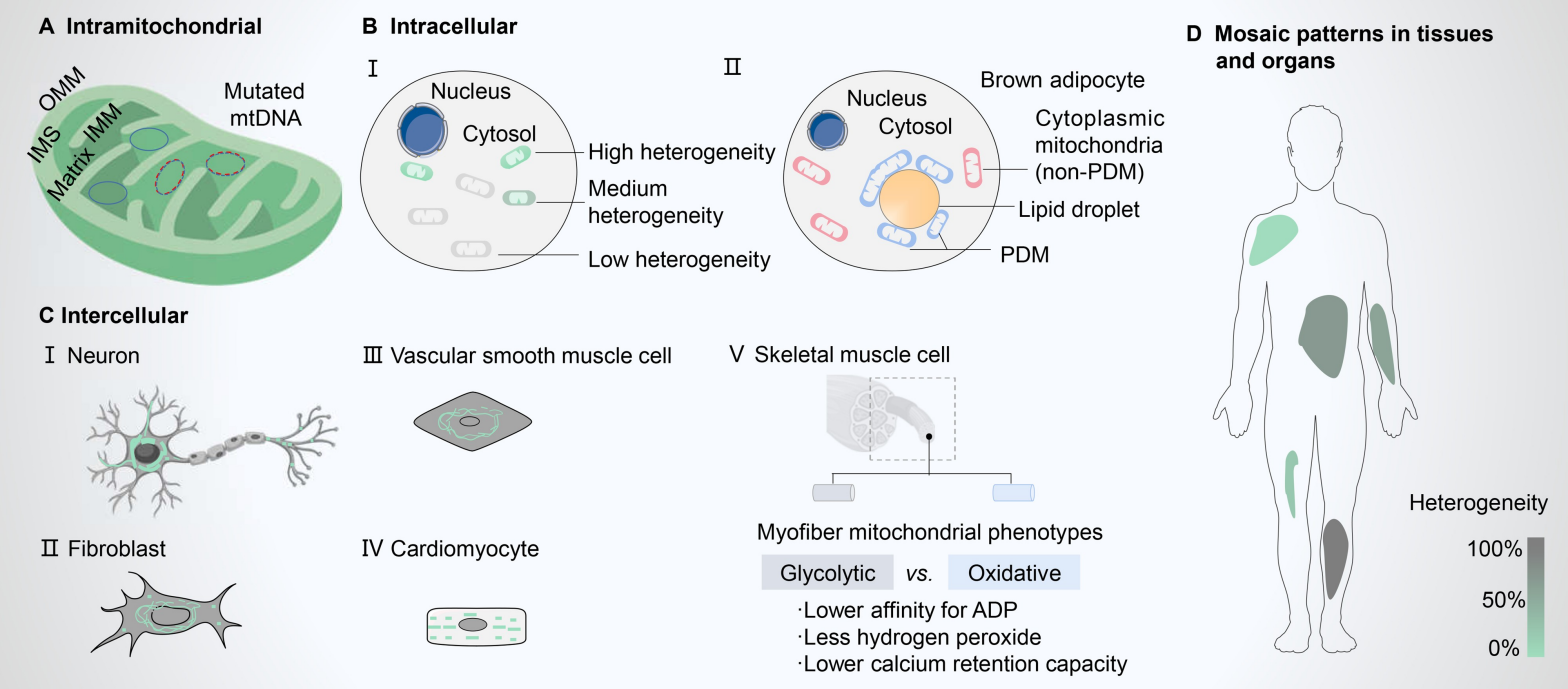
Mitochondrial heterogeneity **A**. A single mitochondrion can carry wild-type mtDNA (blue solid outlines), mutant mtDNA (blue solid + red dashed outlines), or a mix of both. **B**. Diversity of mitochondria in single cells. (Ⅰ) The mitochondria of a cell may have different mutation loads and be of different functions, for example, focusing on energy generation or metabolite synthesis. (Ⅱ) Mouse brown adipocytes contain two major types of mitochondria: PDM and non-PDM. **C**. Cell-type-specific morphology and distribution of mitochondria. (Ⅰ) In neurons, mitochondria are long and form a network at the somatodendritic region. Axonal mitochondria are short rod-like. (Ⅱ) In fibroblasts, mitochondria are distributed evenly, form a network near the nucleus, and are scattered at the cell periphery. (III) In vascular smooth muscle cells, most mitochondria are long tube-like and near the nucleus. (IV) In cells with high energy consumption, such as cardiomyocytes, mitochondria are abundant in the cytoplasm and form a dense network. (Ⅴ) Oxidative and glycolytic muscle fibers coexist in the skeletal muscle. Their mitochondria functionally differ from one another in metabolic pattern. **D**. mtDNA mutations can be seeded during early development or formed during aging and expand into mosaic patterns in tissues and organs. The main elements of the proposed model are available at http://smart.servier.com/ (accessed on 7 September 2023). PDM, peridroplet mitochondria.

Each human mitochondrion contains multiple copies of mtDNA, and the sequence of each molecule can differ, resulting in a heterogeneous mix of mutant and wild-type genomes, a phenomenon known as mtDNA heteroplasmy (**Figure 2**A). Pathogenic mtDNA mutations can lead to significant physiological changes even when present in a heteroplasmic state, where the mutation load is less than 100%. One well-known pathogenic mutation, m.3243A>G (which affects the tRNA^Leu^ encoded by *MT-TL1*), is associated with mitochondrial encephalomyopathy, lactic acidosis, and stroke-like episodes (MELAS) syndrome [5].

mtDNA variants have been recognized as both “inducers” of carcinogenesis and “adaptors” that enable cancer cells to survive and proliferate in diverse environments [6]. However, heteroplasmy is not universally detrimental. Certain mutations within healthy haplotypes have been shown to confer fitness advantages [7].

Molecular diversities contribute to variations in the metabolic patterns of mitochondria. Within a single cell, mitochondria can exhibit distinct characteristics (Figure 2B). For instance, two mitochondrial phenotypes coexist in the cytoplasm of mouse brown adipocytes: peridroplet mitochondria (PDM), which are positioned around lipid droplets, and non-PDM, which are found in the cytoplasmic region [8]. The PDM exhibit higher rates of respiration and ATP synthesis compared to the non-PDM type; however, they possess a lower oxidative capacity for fatty acids (Figure 2B).

Mitochondria also present a wide range of morphological and functional characteristics in response to the specific metabolic demands of different cell types (Figure 2C). The morphology of mitochondria varies across cell types. In neurons, for instance, mitochondria demonstrate significant plasticity in both their structure and function. In the somatodendritic region, these organelles typically form an extended and interconnected network, while in the axon, they tend to exist as smaller, discrete units [2]. In fibroblast cells and cardiomyocytes, mitochondria establish a well-organized network [2] (Figure 2C). Furthermore, cristae are defining features of aerobic mitochondria, and abnormalities in cristae morphology are associated with various physiological and pathological conditions. For example, widened cristae have been observed in hepatic cell lines exposed to hypoxic conditions [9]. In aging mice and fruit flies, cristae undergo widening and develop enveloped voids [10]. Widening of the cristae has also been observed in patients with Leigh syndrome [11]. Additionally, mitochondrial morphology can serve as an indicator of cell death [11]. Notably, mitochondrial fragmentation has been observed during neuronal apoptosis [12]. In cells undergoing ferroptosis, mitochondria exhibit a reduction in size compared to typical morphology, characterized by increased membrane density and reduced cristae formation [13]. In terms of functional differences, mitochondria in cardiomyocytes are specifically optimized for ATP synthesis, while those in the adrenal cortex are specialized for steroidogenesis [14]. In the liver, the predominant mitochondrial types specialize in ketogenesis, serine metabolism, and anaplerosis [15]. In human peripheral blood mononuclear cells (PBMCs), natural variations are observed in mtDNA copy number, citrate synthase activity, and the enzymatic activities of the ETC [16]. Mitochondrial heterogeneity is present in cancer cells, reflecting varying degrees of differentiation [17]. Compared to differentiated glioblastoma multiforme cells, a subpopulation of glioma stem-like cells exhibited significantly lower mitochondrial content and reduced functionality [17]. Even within the same cell type, variations in mitochondrial proteomes can occur. For instance, oxidative and glycolytic cells coexist within skeletal muscle fibers (Figure 2C) [18].

Mitochondrial diversity contributes to mosaicism within tissues and organs, which can be understood in two ways: (1) the coexistence of functionally and morphologically distinct mitochondria, and (2) mtDNA mutations that originate from early germ cells and tend to accumulate specific mutations during the development of various tissues and organs (Figure 2D). For example, mitochondria in mouse brown adipose tissue, heart, liver, and kidney exhibit variations in the expression of respiratory complexes and matrix dehydrogenase activity. These differences, in turn, influence the tissue-specific preference for respiratory substrates [19].

Collectively, there is a relationship between mitochondrial genotype and phenotype. The genotype plays a crucial role in the assembly of oxidative respiratory complexes, which ultimately impacts respiratory capacity. Conversely, the mitochondrial phenotype can influence the genotype by promoting excessive ROS production and impairing mitophagy. As single-cell approaches are increasingly applied in mitochondrial research, the concept of “heterogeneity” can be broadened to encompass both intracellular differences in mtDNA and intercellular variations among distinct cell types.

## Single-cell techniques for the evaluation of mitochondria

Next-generation sequencing (NGS) enables the identification of genetic and functional defects in patients with mitochondrial diseases. The significant advantages of single-cell analysis address the limitations associated with bulk sequencing, such as the masking of mtDNA mutations, differences in gene expression, and dynamic changes within specific cell subpopulations.

**Figure 3:**
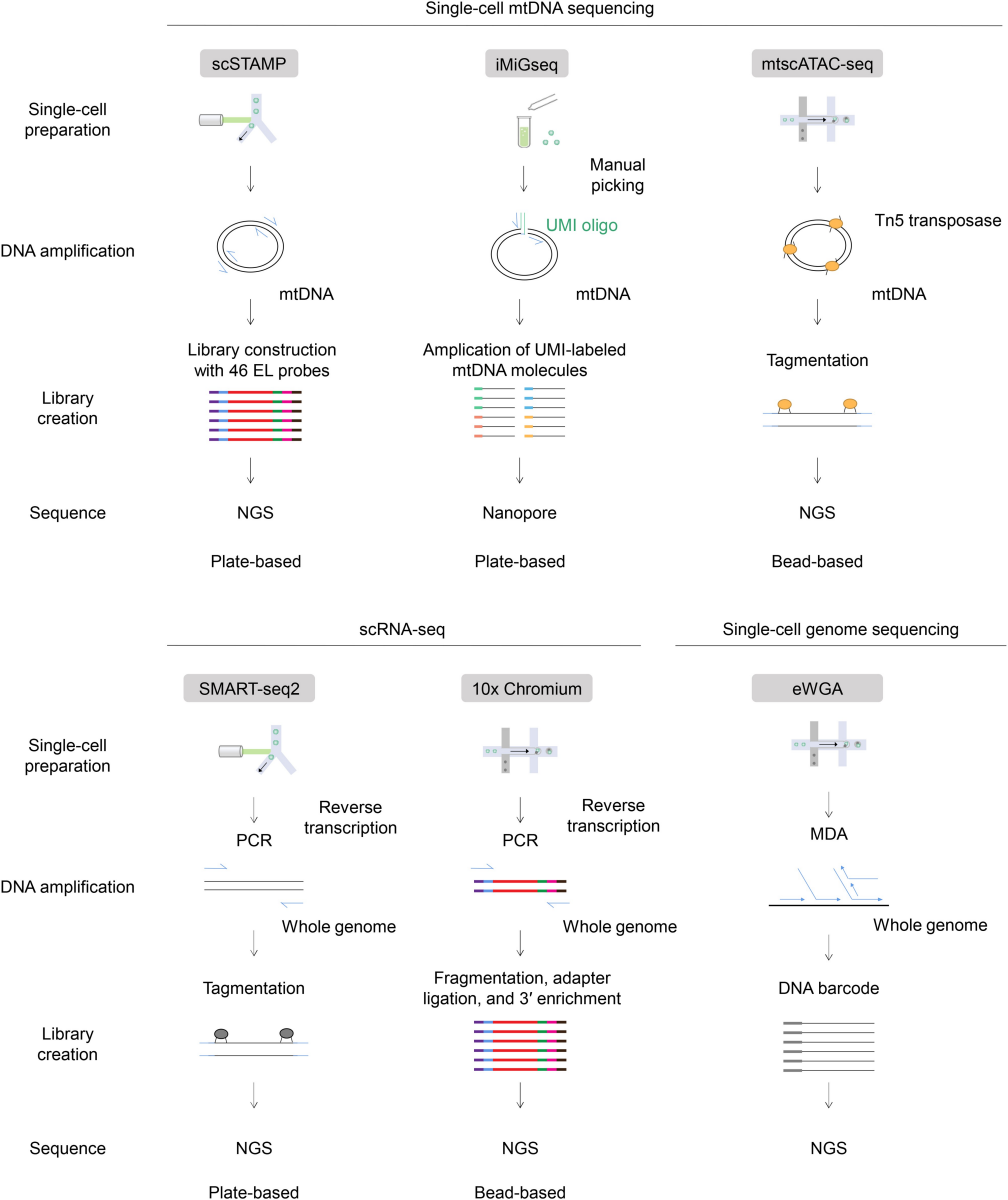
Description of mitochondrial sequencing methods The main process involves dispersing the sample into single cells through manual sorting, flow cytometry, or microfluidic technology. Subsequently, mtDNA is amplified or enriched to construct the library. The selection of sequencing technology is then tailored to the length of the constructed library. EL, extension-ligation; UMI, unique molecular identifier; scmtDNA, single-cell mitochondrial DNA; scSTAMP, single-cell sequencing by targeted amplification of multiplex probes; iMiGseq, individual mitochondrial genome sequencing; mtscATAC-seq, mitochondrial single-cell assay for transposase-accessible chromatin using sequencing; NGS, next-generation sequencing; scRNA-seq, single-cell RNA sequencing; SMART-seq2, switching mechanism at 5′ end of RNA transcript sequencing 2; eWGA, emulsion whole-genome amplification; PCR, polymerase chain reaction; MDA, multiple displacement amplification.

The rapid advancement of single-cell omics has provided new insights and tools for mitochondrial research (**Table 1**). Single-cell isolation can be accomplished through various approaches, including manual picking [20] and high-throughput methods using flow cytometry or microfluidics [21], catering to different requirements and equipment limitations. Researchers have adapted technologies for cell lysis, mtDNA amplification, and data analysis to align with the unique characteristics of mitochondria. By combining multiple sequencing techniques, it is possible to generate an integrated profile of mitochondrial characteristics, offering valuable insights into their roles in various diseases and physiological conditions. Current single-cell methods employed in mitochondrial studies include single-cell mtDNA sequencing, single-cell transcriptome sequencing, and single-cell genome or exome sequencing, as well as other innovative approaches (**Figure 3**).

**Table 1:** Single-cell mitochondrial omics methods by date

Year	Method	Technology	Data types provided	Refs.
2015	scmtDNA-seq	Cycle sequencing	mtDNA	[22,68]
2015	scMito-seq	RCA + NGS	mtDNA	[27]
2019	MitoRACE	Fluorescence microscopy	Mitochondrial energy conversion rate	[39]
2019	scATAC-seq	NGS	mtDNA, chromatin accessibility	[27]
2019	scRNA-seq	NGS	mtDNA, transcriptome	[27]
2020	eWGA	NGS	mtDNA	[33]
2020	MDA	NGS	mtDNA	[33]
2020	Single-cell measurement of mtDNA copy number and heteroplasmy	ddPCR	mtDNA copy number and heteroplasmy	[37]
2021	ASAP-seq	NGS	Chromatin accessibility, protein levels, mtDNA mutations	[28]
2022	scSTAMP	NGS	mtDNA	[21]
2023	iMiGseq	Nanopore	mtDNA	[24]
2023	mtscATAC-seq	NGS	mtDNA, chromatin accessibility, cell state cluster, clonal inference	[26]
2023	Single large-scale mtDNA deletion detection	NGS	Deletions in mtDNA	[41]

*Note*: scmtDNA-seq, single-cell mitochondrial DNA sequencing; scMito-seq, single-cell mitochondrial sequencing; MitoRACE, mitochondrial redox after cyanide experiment; scATAC-seq, single-cell assay for transposase-accessible chromatin using sequencing; scRNA-seq, single-cell RNA sequencing; eWGA, emulsion whole-genome amplification; MDA, multiple displacement amplification; ASAP-seq, ATAC with select antigen profiling by sequencing; scSTAMP, single-cell sequencing by targeted amplification of multiplex probes; iMiGseq, individual mitochondrial genome sequencing; mtscATAC-seq, mitochondrial single-cell assay for transposase-accessible chromatin using sequencing; ddPCR, droplet digital polymerase chain reaction; mtDNA, mitochondrial DNA; NGS, next-generation sequencing; RCA, rolling circle amplification.

### Single-cell mtDNA sequencing

Mitochondrial genomes are highly compact, with approximately 95% of their regions dedicated to coding. Given that mtDNA encodes essential components of the oxidative phosphorylation (OXPHOS) complexes, mutations in mtDNA can result in mitochondrial dysfunction, cellular abnormalities, and even disease. In contrast to the nuclear genome, mtDNA exhibits a significantly higher mutation rate and greater heterogeneity, which requires enhanced sequencing accuracy and advanced data analysis methods. As mtDNA mutations occur randomly within individual cells, they may remain undetectable when analyzing whole tissue samples. Therefore, conducting mtDNA sequencing at the single-cell level is essential for accurate detection.

Current strategies for mtDNA sequencing can be primarily categorized into direct-sequencing methods and indirect-sequencing methods. Mitochondria-targeted sequencing is considered the gold standard for identifying mtDNA mutations. Many mtDNA sequencing techniques utilize a limited number of primers that target specific genes of interest, resulting in the amplification of short fragments (amplicons) that are subsequently sequenced using cycle sequencing [22,23]. We developed a method called single-cell sequencing by targeted amplification of multiplex probes (scSTAMP) to enable whole mtDNA sequencing. In this approach, mtDNA is first amplified as two 8-kb fragments from single-cell lysates to eliminate contamination from nuclear mtDNA-like sequences (NUMTs). The resulting long amplicons are then processed using 46 pairs of extension-ligation probes to create sequencing libraries, which are sequenced with a HiSeq 2500 platform, achieving medium mtDNA coverage of > 100× [21].

However, a limitation of NGS-based mtDNA studies is that rare heteroplasmic variants present at levels below 1% may go undetected due to the background error rate, which ranges from 0.1% to 1%. Individual mitochondrial genome sequencing (iMiGseq) [24] represents a significant advancement by generating long amplicons of full-length mtDNA suitable for nanopore sequencing. This method uniquely labels individual mtDNA molecules with unique molecular identifiers (UMIs) using a single round of primer extension. The sequencing is conducted on long-read Nanopore and PacBio platforms, allowing for unbiased mtDNA analysis. iMiGseq can detect mtDNA single nucleotide variants (SNVs) in cells at levels well below the 1% detection limit. However, the high costs associated with third-generation sequencing technologies may hinder their implementation in high-throughput research projects [24].

In addition to mtDNA-targeted sequencing, another approach involves utilizing off-target reads from exome sequencing, RNA sequencing (RNA-seq), and assay for transposase-accessible chromatin (ATAC) using sequencing (ATAC-seq) to assess mitochondrial variants. Both exome sequencing and RNA-seq can detect mitochondrial variants, but exome sequencing has a lower false discovery rate (FDR) for detecting heteroplasmy compared to RNA-seq [25]. The mitochondrial single-cell ATAC-seq (mtscATAC-seq) enables high-throughput detection of chromatin accessibility and mtDNA. This strategy utilizes mild lysis and permeabilization techniques to facilitate the simultaneous transposition of nuclear accessible chromatin and mtDNA by Tn5 transposase [26]. The mtscATAC-seq generates a library containing at least 1000 mtDNA fragments in 80%–90% of the cells, with a mean coverage of 28.9× across the mtDNA genome. Single-cell assessments of mtDNA mutations using single-cell RNA-seq (scRNA-seq) or single-cell ATAC-seq (scATAC-seq) data can simultaneously yield insights into lineage tracing [27]. If there is a need to study cell states, indirect-sequencing methods can be cost-effective. However, as a “by-product”, only about 20% of the total scATAC-seq data from a single cell comprise mtDNA reads, which can result in insufficient depth of mtDNA coverage [21].

In the multi-omics sequencing method known as ATAC with select antigen profiling by sequencing (ASAP-seq), mtDNA is preserved within droplets containing single cells. This technique employs a bridging approach that facilitates the simultaneous detection of chromatin accessibility, protein levels, and mtDNA mutations [28].

### Single-cell transcriptome sequencing

Single-cell transcriptome sequencing is extensively utilized to identify and characterize various cell types, states, and lineages. The conventional scRNA-seq pipeline can analyze differentially expressed mitochondrial genes and uncover cell types with distinct mitochondrial profiles. In ascending thoracic aortic aneurysm (ATAA) tissues, single-cell transcriptome analysis reveals differences in OXPHOS gene expression among individual cells and highlights the role of mitochondria in ATAA formation and progression [29]. Another scRNA-seq study of mouse epididymal cells showed higher expression levels of mitochondrial genes in the corpus and cauda epididymis compared to the caput region. The elevated number of mitochondria and mitochondrial transcription in the epididymis may provide adequate energy production to support its functional requirements [30]. Given that mtDNA is nearly entirely transcribed, mitochondrial mutations can be detected through scRNA-seq, serving as a natural barcode for lineage tracing. Researchers utilized the “discarded” mtDNA data from scRNA-seq and scATAC-seq to perform lineage tracing. In a comparative analysis of scRNA-seq methods, six different techniques were applied to mouse embryonic stem cells to evaluate their effectiveness [27]. The results indicate that scRNA-seq data generated from plate-based approaches exhibited superior mitochondrial genome coverage compared to droplet-based methods. Consequently, the plate-based switching mechanism at 5′ end of RNA transcript sequencing 2 (SMART-seq2) technique has broader applications in subsequent studies [31].

RNA-seq data for mitochondrial genes can be selectively filtered to evaluate mtDNA transcript levels. An scRNA-seq analysis revealed that mtDNA transcript levels were higher in pancreatic beta cells than in alpha cells in both healthy individuals and patients with diabetes [32]. mtDNA variations can be analyzed in conjunction with mitochondrial gene expression levels to identify new cell types. For example, pancreatic beta cells within an individual can be categorized into subpopulations based on their mitochondrial signatures, which include the mitochondrial mutational repertoire and the transcript levels of OXPHOS components encoded by both mtDNA and nuclear DNA [32]. scRNA-seq facilitates a detailed analysis of cell subtypes, providing a more comprehensive understanding of diseases associated with mitochondrial dysfunction.

While current scRNA-seq techniques are effective in revealing cell states and mtDNA mutations, there are several ways to enhance their application in mitochondrial research. Firstly, high-throughput scRNA-seq methods, such as droplet-based approaches, often exhibit limited coverage of mtDNA [27]. Employing methods to enrich mitochondrial transcripts could significantly improve the effectiveness of this approach [27]. Secondly, combining mtDNA mutations with nucleogenic SNVs, copy number variations (CNVs), and microsatellites can enhance the accuracy of lineage tracing. Lastly, improving the accuracy of mtDNA data necessitates the refinement of filtering and mapping strategies [25].

### Single-cell genome or exome sequencing

Whole-genome and whole-exome sequencing are essential tools for investigating mtDNA in extensive population studies. Utilizing a computational pipeline to filter mtDNA sequencing data from existing datasets proves to be a cost-effective strategy [33]. The pipeline consists of three primary stages: (1) preprocessing raw sequencing data into FASTQ format and eliminating low-quality reads; (2) mapping the filtered reads to the reference genome to extract mtDNA reads; and (3) detecting and annotating mtDNA variants. A comparative analysis of mtDNA genome reconstruction efficiency demonstrated that emulsion whole-genome amplification (eWGA) and multiple displacement amplification (MDA) methods, outperformed other methods by utilizing low FDR genome sequencing strategies with phi29, a high-fidelity polymerase [33].

Once the whole-genome libraries have been sequenced, mitochondrial variants are identified. Historically, these data have often been regarded as “noise” in genome or exome sequencing. However, they can be applied to studies involving tissues, such as normal and tumor cells, to investigate somatic mutations and their contributions to tumorigenesis [34]. Single-cell genome sequencing can be employed to investigate the mitochondrial genomes of protists, which are unicellular organisms. Recently, the genomes of retarians, including foraminifera and radiolaria, have been sequenced. These findings enhance our understanding of mitochondrial genome evolution and expand the mtDNA sequencing databases [35].

In addition to sequencing methods, alignment strategies significantly influence the validation rate and FDR of heteroplasmies. Simultaneously aligning reads to both nuclear and mitochondrial reference genomes or directly aligning reads to the mitochondrial reference genome yields high-quality results. Conversely, aligning reads to the nuclear genome first and then to the mitochondrial genome leads to suboptimal performance [25]. Aligning reads to both nuclear and mitochondrial reference genomes helps avoid interference from pseudogenes or homologous sequences present in the nuclear DNA [34].

### Other single-cell technologies

Several evaluation indices are available for assessing mitochondrial functions, and corresponding strategies at single-cell resolution have been developed. Droplet digital polymerase chain reaction (ddPCR) utilizing microwell chips and microfluidic techniques can effectively identify rare mutations within samples [36]. Single-cell ddPCR (sc-ddPCR) allows for analysis within a single droplet that encapsulates an intact single cell. This method has been developed to investigate intercellular and intracellular heteroplasmy, as well as to quantify mtDNA copy number [37]. The benefits of sc-ddPCR include its high sensitivity and the ability to accurately quantify absolute mtDNA copy numbers.

The proliferation heterogeneity of individual yeast cells was quantified using a high-throughput microscopy assay. It was found that mitochondrial membrane potential, rather than the quantity of mitochondria, varies among single yeast cells and is associated with intercellular heterogeneity in proliferation, mutations, stress tolerance, and drug resistance [38]. Cells exhibiting different mitochondrial phenotypes can be sorted using flow cytometry based on mitochondrial membrane potential, mass, or ROS levels. Following direct lysis or monoclonal formation, the genetic material obtained from a single cell can be utilized for various omics experiments and integrated with corresponding cell phenotypes [38]. Additionally, mitochondrial redox after cyanide experiment (mitoRACE) can evaluate energy metabolism by analyzing nicotinamide adenine dinucleotide hydride (NADH) autofluorescence kinetics at single-cell or subcellular resolution within intact cells. Metabolic imaging of NADH provides insight into mitochondrial energy status and is a crucial component of mitochondrial phenomics [39].

Multi-omics methodologies that operate at single-cell and spatial resolution facilitate a thorough investigation of the intermolecular dynamics between gene variation, regulation, and expression at both the transcriptome and proteome levels within the same single cell throughout development, aging, and disease. A significant challenge in advancing single-cell omics research related to mitochondria lies in the integration and interpretation of a vast array of single-modality data. There is an urgent need for unified sequencing guidelines and data integration software to address this issue.

### Computational approaches tailored for mitochondrial omics analysis

Mitochondria are characterized by their heterogeneity and a notably high mutation rate of mtDNA, which is significantly smaller than the nuclear genome. As a result, a key challenge in single-cell mitochondrial sequencing is distinguishing genuine signals from background noise. In addition to advancements in single-cell sorting techniques and sequencing technology, computational approaches are essential for filtering mitochondrial data. Below are some recent developments in computational strategies aimed at addressing this issue.

Several computational tools have been developed to identify mtDNA variants amidst the “noise” present in sequencing data. Mixture Modeling of Mitochondrial Mutations (MQuad) [40] is a method designed to detect informative mtDNA variants and can be integrated with tools like cellSNP and vireoSNP, which offer an analysis framework for mtDNA genotyping and clonal reconstruction. The mitochondrial genome analysis toolkit (mgatk) is capable of identifying low-frequency somatic mtDNA mutations (ranging from 0.005% to 0.01%) within the overall mtscATAC-seq data [26].

mgatk-del is a module within mgatk specifically designed to quantify heteroplasmy associated with deletions in mtDNA. This tool filters base-level breakpoints in sequencing reads to identify deletion junctions. The heteroplasmy of these deletions is estimated based on the ratio of reads that align with the deleted junction sequence [41].

## Single-cell mitochondrial dysfunction and heterogeneity in diseases and aging

**Figure 4:**
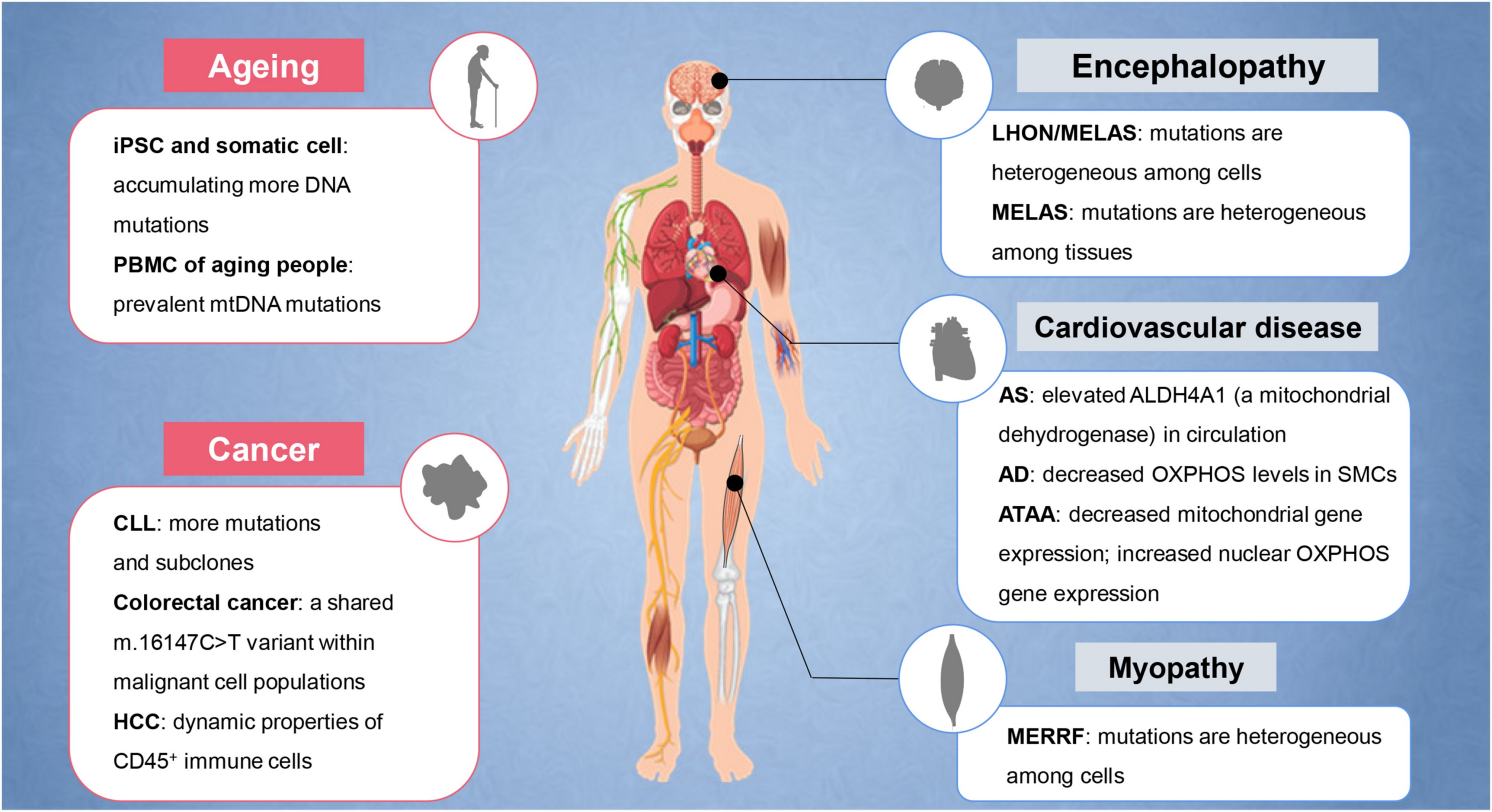
Single-cell mitochondria dysfunction/heterogeneity in diseases The main elements of the proposed model are available at http://smart.servier.com/ (accessed on 7 September 2023). AD, aortic dissection; AS, atherosclerosis; ATAA, ascending thoracic aortic aneurysm; CLL, chronic lymphocytic leukemia; HCC, hepatocellular carcinoma; iPSC, induced pluripotent stem cell; PBMC, peripheral blood mononuclear cell; LHON, Leber hereditary optic neuropathy; MELAS, mitochondrial encephalomyopathy, lactic acidosis, and stroke-like episodes; ALDH4A1, aldehyde dehydrogenase 4 family, member A1; OXPHOS, oxidative phosphorylation; SMC, smooth muscle cell; MERRF, myoclonic epilepsy with ragged-red fibers.

Mitochondrial disorders can affect any organ, can manifest at any age, and are rarely curable. Beyond established mitochondrial diseases, mitochondrial dysfunction and heterogeneity are also present in metabolic disorders, including cancer and cardiovascular diseases (CVDs), as well as in natural physiological processes such as aging. Examining the genotype and phenotype of mitochondria at the single-cell level in these contexts can yield valuable insights into the mechanisms underlying disease and development (**Figure 4**).

### Mitochondrial diseases

Certain mitochondrial diseases have been linked to mtDNA mutations. A mutation load of 60%–80% typically results in limited mitochondrial dysfunction and is associated with mild symptoms that may onset during adolescence or adulthood. Conversely, a mutation load exceeding 90% is generally indicative of significant mitochondrial impairment, often accompanied by severe disorders [42].

Leber hereditary optic neuropathy (LHON) is associated with high levels of mtDNA mutations. In bulk assays, the average heteroplasmy observed in fibroblasts derived from LHON patients was found to be 100%. In contrast, single-cell assays revealed that approximately 98.3% of LHON samples displayed mutant mtDNA in a homoplasmic state, while the remaining samples exhibited a mixture of mutant and healthy mtDNA [37]. Myoclonus epilepsy with red ragged fibers (MERRF) is a mitochondrial disorder primarily caused by the m.8344A>G mutation, which disrupts transfer RNA (tRNA) function. Bulk ATAC-seq analysis of lymphoblastoid cells from a MERRF patient estimated the population heteroplasmy for the m.8344A>G mutation to be approximately 44%. In contrast, single-cell sequencing revealed a median heteroplasmy of 38% for the m.8344A>G allele, aligning with the findings from the bulk ATAC-seq data. However, the heteroplasmy among individual cells varied widely, ranging from 0% to 100% [43].

The m.3243A>G mutation affects mitochondrial tRNA^Leu^, and presents a broad phenotypic spectrum, which includes symptoms such as deafness, diabetes, gastrointestinal dysmotility, epilepsy, and stroke-like episodes. scATAC-seq analysis revealed a T cell-specific reduction in m.3243A>G heteroplasmy when compared to the aggregate PBMCs from patients diagnosed with MELAS [44]. In the retina of a patient with MELAS [45], the load of the m.3243A>G mutation varied among different cell types. Notably, the heteroplasmy of m.3243A>G was found to be higher in the neural retina and retinal pigment epithelium (RPE) than in the choroidal endothelium. Single-cell technologies are instrumental in identifying cell types with elevated mutation loads, helping to determine which cells are more vulnerable to the effects of the mutation.

mtDNA deletions have been observed in patients with congenital anomalies. In the PBMCs of these patients, various types of T cells demonstrate different tolerance to pathogenic mtDNA deletions within the mitochondrial genome. Furthermore, there is evidence of dynamic purifying selection occurring within human immune cells [41].

### CVDs

CVDs rank as one of the leading causes of mortality globally, with atherosclerosis being the primary trigger for adverse cardiovascular events [46,47]. Single-cell analyses have indicated that ALDH4A1, a mitochondrial dehydrogenase, serves as a target antigen for antibodies expressed by B lymphocytes in the context of atherosclerosis [48]. Circulating levels of ALDH4A1 are elevated in both mouse models and patients with atherosclerosis [48]. Anti-ALDH4A1 antibodies have the potential to slow the progression of atherosclerosis and may offer therapeutic benefits in the context of CVDs.

In other cardiovascular conditions, such as hereditary and non-hereditary aortic aneurysms and dissections, mitochondrial dysfunction in vascular smooth muscle cells (VSMCs) has been established as a contributing factor to the development of aortic lesions [49,50]. Cells within ATAA tissues also display mitochondrial dysfunction. Further analysis through single-cell transcriptomics uncovered a significant decrease in mitochondrial gene expression, coupled with increased expression of nuclear-encoded OXPHOS elements across 24 out of 40 cell types present in ATAA tissues [29]. Our scRNA-seq study of the human dissected aorta indicates that the reduction in mitochondrial OXPHOS within specific contractile smooth muscle cells (SMCs) is a critical factor driving phenotypic transformation. The expansion of classical myofibroblasts and lipofibroblasts can lead to aortic dissection and rupture. Through both *in vitro* and *in vivo* experiments, we confirmed that targeting the TNF–OXPHOS–AP-1 axis represents a promising strategy for managing aortic dissection [51].

In summary, single-cell research has revealed critical pathogenic cell populations and mitochondria-related signaling pathways involved in CVDs. Targeting mitochondrial function presents a promising therapeutic approach for managing CVDs.

### Cancer

mtDNA mutations are prevalent in cancers. Tumors comprise heterogeneous cell populations, each potentially harboring distinct mtDNA genotypes. These mtDNA mutations can be lineage-specific or cancer-specific, with the majority of somatic mutations existing in a heteroplasmic state [52]. As a result, quantifying heteroplasmy levels in tumors using bulk sequencing methods can be complicated by the presence of distinct cell populations. However, new single-cell sequencing techniques offer the capability to accurately quantify mtDNA heteroplasmy in diseases characterized by highly heterogeneous foci, such as cancer.

Somatic mtDNA mutations are hypothesized to emerge during tumorigenesis, allowing for the tracking of genetic subclones to analyze tumor heterogeneity. In human malignancies such as chronic lymphocytic leukemia (CLL), monoclonal B cell malignancy is a significant characteristic. High-quality scRNA-seq has uncovered a greater number of mutations and subclones, including a specific cluster of leukemic cells characterized by the m.12067C>T mutation. In human colorectal cancer, the mtscATAC-seq platform has identified epithelial cells and distinct immune cells as major cellular populations [43]. The results showed that a homoplasmic m.16147C>T variant is present across the major malignant cell populations. Specific mutations may serve as potential markers for distinguishing between malignant and nonmalignant cells. Additionally, SMART-seq2 was utilized in a transcriptomic study of CD45^+^ immune cells to analyze the immune microenvironment in hepatocellular carcinoma [31]. The mtDNA mutations identified through scRNA-seq were utilized for lineage tracing, revealing that many macrophages present in tumors and ascites share common lineages. Within the tumor microenvironment, dendritic cells (DCs) have traditionally been classified into 3 subsets, including conventional DC (cDC), cDC1, and cDC2. This study identified a novel population of DCs characterized by *LAMP3* expression (*LAMP3*^+^ DCs), which exhibited broad regulatory functions in modulating lymphocyte activity. Furthermore, single-cell whole-genome sequencing revealed that the coregulation of nuclear and mitochondrial genomes drives dynamic changes in mtDNA within individual tumor cells [53].

### Aging

Aging cells, including induced pluripotent stem cells (iPSCs) and somatic cells [54], accumulate a higher number of DNA mutations and demonstrate increased instability compared to normal cells. iPSCs derived from older individuals possess somatic genomic variations and distinct epigenetic signatures [55,56]. Single-cell mitochondrial sequencing was performed to detect mtDNA mutations in iPSCs derived from individuals aged 24 to 72 years [57]. The results indicate that nonsynonymous and RNA-coding gene mutations are present in individual cells but are not detectable in whole tissue analyses. These mutations have the potential to induce respiratory defects and warrant further investigation.

Similar scenarios are observed in highly differentiated cells such as PBMCs. A single-cell mitochondrial sequencing study of B lymphocytes and monocytes from a 76-year-old female identified prevalent mtDNA mutations with functional significance [21]. The mtDNA mutations were found in over half of the cells, with variant allele frequencies (VAFs) exceeding 20%. Additionally, more than 50% of the nonsynonymous mutations were classified as highly pathogenic.

Given the potential influence of mtDNA mutations on the metabolic status of cells, detecting these mutations in iPSCs and somatic cells obtained from elderly patients holds significant relevance for clinical applications and disease treatment.

The limitations of bulk sequencing, especially its inability to detect rare senescent cell populations (< 5% of tissue cells), have hindered research on cellular senescence. Single-cell approaches have now uncovered mitochondrial dysfunction as a critical link between cellular senescence, inflammation, and failures in tissue regeneration. This insight paves the way for targeted interventions, such as neutralizing CD36 to reduce inflammation and enhancing mitophagy [58].

## Potential applications for evaluating mitochondria in a single cell

The advancement of technologies alongside findings from the lab aims to facilitate practical applications for enhancing human health. Mitochondria exhibit significant heterogeneity, and single-cell omics technologies have markedly increased accuracy and sensitivity compared to bulk sequencing. These technologies enable the identification of mitochondrial gene mutations and differences at transcript levels within specific cell types that are directly linked to diseases. On one hand, the cell types and mutations identified through single-cell methodologies can serve as valuable markers in clinical diagnosis and genetic counseling for various conditions. On the other hand, a deeper understanding of disease mechanisms can aid in the development of targeted therapies.

### Exploration of pathogenic mechanisms of disease

The primary advantage of single-cell mitochondrial sequencing methods lies in their capability to analyze the mitochondrial genotype and phenotype of each cell subpopulation. This integrated information can aid in the identification of pathogenic mtDNA variants and key cell types involved in human diseases. Future therapies may focus on specific cell types or mitochondria rather than targeting all cells or all mitochondria within a cell. This increased precision could enhance safety and reduce costs in clinical trials, especially considering that adeno-associated viruses (AAVs) are among the most commonly used vectors for targeted delivery. Additionally, single-cell omics can provide insights into fundamental aspects of mitochondrial genetics. A method for detecting mtDNA heteroplasmy, known as single-cell combinatorial indexing leveraged to interrogate targeted expression (SCI-LITE), has also demonstrated that shifts in heteroplasmy involving synonymous variants are driven by cell-level selection that is entirely dependent on the cellular environment [59].

### Early screening and disease prevention

Mitochondrial mutations gradually accumulate in various diseases, with the severity of symptoms correlating to mutation loads. Therefore, identifying disease-related mutations and implementing timely interventions at an early stage can help prevent disease progression. Single-cell sequencing methods are capable of detecting low-level mutations that bulk sequencing may overlook, as well as mutations that are specific to particular cell types. These attributes position single-cell mitochondrial sequencing as a highly promising tool for early screening in mitochondrial disorders. For instance, healthy human oocytes and blastoids [24] have been found to contain low levels of rare heteroplasmic variants. Maternal mitochondria serve as the sole source of mtDNA in offspring, and the heterogeneous mtDNA mutations inherited from oocytes often contribute to metabolic disorders. Additionally, as the mutation load increases, these mutations are also linked to late-onset diseases.

### Mitochondrial drug development

Single-cell research can facilitate the development of mitochondrial drugs in three key ways: (1) enhancing understanding of etiology and pathogenesis; (2) assessing treatment effects; and (3) evaluating safety. The single-cell sequencing technology enables the analysis of individual cells, uncovering mutations that may be missed in bulk sequencing. Typically, it is a specific subpopulation of cells that is responsible for the observed symptoms [17]. Identifying the underlying causes and specific targets can enhance treatment outcomes and minimize adverse reactions, particularly in gene therapies.

Mitochondrial drugs may also provide benefits for patients with autosomal genetic diseases. Many autosomal genetic disorders involve highly complex gene interactions that are challenging to treat or cure with single-gene therapies. Enhancing mitochondrial function has been shown to serve as an alternative approach for individuals suffering from energy deficiencies. An example of this is Friedreich’s ataxia, a hereditary neurological disorder caused by the abnormal expansion of GAA repeats in the non-coding region of the frataxin gene. Frataxin plays a crucial role in mitochondrial energy production. In a clinical trial, the NRF2 activator omaveloxolone demonstrated the ability to restore mitochondrial function and improve scores on the modified Friedreich’s ataxia rating scale [60]. Skyclarys (omaveloxolone) is the only drug approved by the US Food and Drug Administration (FDA) for patients with Friedreich’s ataxia. Given the potent effects of NRF2, adverse reactions are a possibility. Therefore, it is essential to strike a balance between safety and effective enhancement of mitochondrial functions in the affected nerve fibers within the spinal cord and peripheral nerves. In this regard, single-cell mitochondrial sequencing will serve as a powerful tool for assessment.

### Lineage tracing

Lineage tracing allows for the determination of the fate of individual cells. Many somatic mtDNA mutations can be stably inherited, which enhances the feasibility of lineage tracing studies. In *in vivo* experiments, engineered genetic labels such as the Cre-loxP system [61], fluorescent reporter genes, and clustered regularly interspaced short palindromic repeats (CRISPR)/CRISPR-associated protein 9 (Cas9)-induced genetic scars [62] can be applied to individual cells, serving as heritable markers. In human studies, the most commonly employed approach for detecting nuclear somatic mutations is whole-genome sequencing, which is often costly and associated with high error rates. An inherent and natural alternative lies in mtDNA variation. mtDNA has a considerably high mutation rate due to its exposure to ROS, random genetic drift, and the absence of precise repair mechanisms. The mutation rate for mtDNA is approximately 10 to 100 times higher than that of nuclear DNA mutations, and these mutations can be stably inherited by daughter cells. Additionally, the large copy number of mtDNA in a single cell, ranging from hundreds to tens of thousands [42], facilitates the acquisition of material for single-cell studies. Detection methods for mtDNA variations at the single-cell level have been well established and can be integrated with cell state measurements, including scATAC-seq and scRNA-seq [27] or further analysis. A subsequent analytical method that requires only scRNA-seq or scDNA-seq data has also been developed, featuring accurate pedigree analysis capabilities [40,63].

There are potential limitations to consider. The possibility of selective mitochondrial inheritance or intercellular mitochondrial transfer may impact the accuracy of inferred lineages. However, some researchers have proposed that horizontal transfer may not substantially affect the analysis and does not occur universally between cells [27,64]. However, the high cost and low throughput associated with the modified SMART-seq2 sequencing technology, which is commonly used in current studies, limit the application of mtDNA as a natural genetic cell barcode for clonal tracking. A promising alternative to mitigate costs and enhance coverage is the adoption of targeted mtDNA sequencing methods.

### Genetic counseling in mtDNA mutation-related diseases

For families at risk of genetic diseases, genetic counseling is recommended to understand the inheritance pattern of the disease and to minimize the risk in offspring. In contrast to autosomal diseases, mtDNA mutation-related diseases follow a maternal inheritance pattern. A homozygous pathogenic mutation in the ovum can be transmitted to the zygote. The situation becomes more complex when considering heteroplasmic mutations due to the phenomenon of mitochondrial bottleneck during oogenesis. Unequal partitioning of mitochondria during cell division can result in varying levels of heteroplasmy in mature oocytes. Oocytes with a lower mutation load are more likely to develop into offspring without mitochondrial diseases. By conducting single-cell assessments of oocytes, researchers may assist mothers with heteroplasmic mtDNA mutations by filtering germ cells with lower mutation loads. Given the observation of entirely healthy mitochondria in traditional homozygous mitochondrial diseases [37], there is a possibility of obtaining healthy oocytes even from adult patients. Although avoiding injury to the zygote during mitochondrial assessment sampling poses a significant challenge, this approach remains a viable method for reducing the risk of offspring inheriting mitochondrial diseases.

### Preclinical safety assessment

Among the new discoveries made using single-cell mtDNA sequencing, a significant finding is that mtDNA mutations are present not only in disordered cells but also in unsuspected ones. One potential application for this information is in the quality control of chimeric antigen receptor (CAR)-T cells. Early exhaustion of T cells represents a major limitation to the efficacy and broader application of CAR-T cell therapies [65]. The costimulatory domains and initial phenotypes of CAR-T cells significantly influence their persistence and cytotoxicity. Mitochondrial metabolism plays a crucial role in the development or failure of T cell memory [65]. An enhancement in mitochondrial spare respiratory capacity has been shown to correlate with an extended lifespan of T cells [66]. In preclinical models, T cells with a dominant OXPHOS metabolism demonstrate a longer lifespan following adoptive transfer and exhibit improved antitumor functionality. Therefore, evaluating mitochondrial quality prior to CAR-T cell therapy could potentially enhance treatment efficacy [67]. Importantly, a targeted single-cell mtDNA sequencing method known as iMiGseq, can be used for safety evaluation of gene editing tools [24]. On-target or off-target edits frequently produce low-frequency variants that are challenging to detect using NGS. Results from iMiGseq revealed unintended shifts in mtDNA heteroplasmy following mitochondrially targeted transcription activator-like effector nuclease (mitoTALEN) editing, whereas no significant off-target mutations were observed in mtDNA base editing mediated by the DddA-derived cytosine base editor (DdCBE) system.

## Conclusion and perspectives

Remarkable advancements have been made in mitochondrial research at the single-cell level over the past few decades, encompassing improvements in sequencing technologies, data analysis methods, and our fundamental understanding of mitochondrial biology across diverse physiological conditions. The key findings from the application of single-cell mitochondrial sequencing are as follows: (1) mtDNA heterogeneity ranges widely from 0% to 100% within individual cells, whereas bulk sequencing provides only a median value; (2) certain types of mitochondria or cells are specifically associated with particular disease phenotypes, offering potential targets for precision medicine; and (3) mtDNA mutations with low heterogeneity are prevalent in “healthy” cells. These unexpected mutations necessitate thorough safety evaluations before the implementation of stem cell transplantation, gene editing, and other novel clinical therapies.

Similar to other single-cell library construction methods, single-cell mitochondrial sequencing necessitates the use of fresh samples to isolate and sort single, intact cells, which significantly limits the applicability of this approach. Additionally, the spatial organization of the genome influences cellular function. For example, within a single neuron, mitochondria located in neurites exhibit higher energetic flux than those in the cell body [39]. Given the intracellular heterogeneity of mitochondria, assessments at the single-cell level may be insufficient to fully evaluate mitochondrial functions and elucidate the pathogenic mechanisms underlying complex diseases. To address this limitation, methods have been developed to achieve high-resolution DNA sequencing *in situ* within intact biological samples [68].

Sequencing libraries can be constructed and sequenced *in situ* following sample fixation. After this, the samples are transferred into an imaging buffer for immunostaining and immunofluorescence. This approach eliminates the need for mitochondrial isolation. Consequently, the sequencing data of mtDNA can be computationally extracted and integrated with images obtained from fluorescence probes that are resistant to cell fixation.

Single-cell omics technologies provide researchers with enhanced insights into mitochondrial diseases. Once the pathogenic mechanisms are identified, targeted strategies can be employed to improve mitochondrial function. For instance, functional proteins (or those with similar functions) can be transported to replace malfunctioning proteins, or small molecules can be utilized to mitigate adverse effects, such as excessive ROS, suppressed mitophagy, and reduced energy production. However, these therapeutic approaches are often classified as “broad-spectrum”.

An example of a targeted therapeutic approach is Lumevoq (GS010), a gene therapy developed for the treatment of LHON, particularly triggered by *ND4* mutations. Lumevoq utilizes AAV2 to introduce a correct copy of the *ND4* gene into the cell nucleus. Although AAV2 is commonly employed for delivery to retinal ganglion cells (RGCs), where LHON symptoms primarily manifest, this serotype may also infect other ocular cells (such as RPE and photoreceptor cells) that may have normal *ND4* expression [65]. Lumevoq also exhibited a contralateral effect during clinical trials, raising concerns regarding the drug’s overall effectiveness and safety [64].

Small-molecule therapies currently represent the most commonly used treatments for mitochondrial dysfunction, including antioxidants (*e.g.*, MitoQ) and mitophagy activators (*e.g.*, urolithin A) [69,70]. However, patients with the same type of mitochondrial disease often display varied phenotypes due to the combined influence of modifying genes, the threshold effect, and environmental factors [71]. Numerous clinical trials have evaluated the efficacy of small molecules in treating mitochondrial diseases (**Table 2**) [72], but only a limited number of drugs (*e.g.*, elamipretide and idebenone) have demonstrated beneficial effects [73,74].

**Table 2:** Summary of small molecules’ effects on mitochondrial functions and the results of related clinical trials

Compound	Mechanisms of action	Adapted disease/syndrome	Clinical trial	Result
**Increasing mitochondrial mass and NAD^+^ levels**				
Bezafibrate	PPAR agonist; increase mitochondrial biogenesis	Mitochondrial myopathy	NCT02398201: a study of bezafibrate in mitochondrial myopathy	Completed; results awaited
Resveratrol	Activator of AMPK and SIRT1; increase mitochondrial biogenesis	Mitochondrial myopathies and skeletal muscle fatty acid oxidation disorders	NCT03728777: resveratrol supplementation in patients with mitochondrial myopathies and skeletal muscle fatty acid oxidation disorders	Ineffective in improving exercise capacity in adults with mitochondrial myopathies
Omaveloxolone (RTA-408)	Potent NRF2 activator; inhibit NF-κB; induce mitochondrial biogenesis and an antioxidant and anti-inflammatory phenotype	Mitochondrial myopathy	NCT02255422: RTA-408 capsules in patients with mitochondrial myopathy — MOTOR	Failed to improve the primary outcome measure (peak exercise workload) or the secondary outcome (6MWT) at week 12 in patients with mitochondrial myopathy
Niacin	NAD^+^ precursor, activating mitochondrial biogenesis pathway	Mitochondrial myopathy	NCT03973203: niacin supplementation in healthy controls and mitochondrial myopathy patients (NiaMIT)	Completed; results awaited
Nicotinamide riboside	NAD^+^ precursor, activating mitochondrial biogenesis pathway	Skeletal muscle mitochondrial function in obese men	NCT02303483: the effects of vitamin B3 on substrate metabolism, insulin sensitivity, and body composition in obese men	No significant impact on skeletal muscle mitochondrial functions and morphology
Acipimox (5-carboxy-2-methylpyrazine 1-oxide)	NAD^+^ precursor, activating mitochondrial biogenesis pathway	To improve the muscle mitochondrial function in obesity	NCT01488409: the effects of acipimox on mitochondrial function in obesity	Failed to improve the muscle mitochondrial function
**Reducing oxidative stress**				
Sonlicromanol (KH176)	ROS scavenger	Genetically confirmed mitochondrial disease and motor symptoms	NCT04846036: the KHENERGYC study	Recruiting
MitoQ	ROS scavenger	Aging	NCT02597023: the efficacy of oral MitoQ supplementation for improving physiological function in middle-aged and older adults	Elevating vascular function
Cysteamine bitartrate (RP103)	Antioxidant	Inherited mitochondrial disease	NCT02023866: an open-label, dose-escalating study assessing the safety, tolerability, and efficacy of RP103 in mitochondrial disease	Lack of efficacy
**Improving mitochondrial function**				
Elamipretide (MTP-131)	Protecting cardiolipin and guarding normal bioenergetics	Mitochondrial myopathy	NCT02367014: safety, tolerability, and efficacy of MTP-131 for the treatment of mitochondrial myopathy (MMPOWER)	Increase in distance walked on the 6MWT
Idebenone	An analog of CoQ10, electron carrier	LHON	NCT00747487: a study to assess the efficacy, safety, and tolerability of idebenone in the treatment of LHON (RHODOS)	Beneficial effect on restoring or maintaining visual function
**Restoring mtDNA homeostasis**				
Urolithin A (AMAZ-02)	Mitophagy activator	Aging	NCT03464500: the effects of AMAZ-02 on exercise tolerance in healthy, overweight middle-aged subjects (ATLAS Trial)	Improvements in muscle strength

*Note*: PPAR, peroxisome proliferator-activated receptor; AMPK, adenosine monophosphate-activated protein kinase; SIRT1, Sirtuin 1; 6MWT, 6-min walk test; CoQ10, ubiquinone; MitoQ, mitoquinone; NAD, nicotinamide adenine dinucleotide; NF-κB, nuclear factor kappa-B; NRF2, Nuclear factor erythroid-2-related factor 2; ROS, reactive oxygen species; LHON, Leber hereditary optic neuropathy.

Findings from single-cell mitochondrial omics indicate that abnormalities in mitochondrial function associated with diseases may occur in specific cell types while remaining normal in others. This specificity may explain why “broad-spectrum” function enhancement is often ineffective or can lead to side effects. Strategies for mitochondrial targeting — such as chemical modification of molecules to enhance lipophilicity and positive charge, utilization of mitochondrial targeting signal (MTS) peptides, and stimulus-responsive approaches (*e.g.*, ROS) — enable precise delivery of therapeutic small molecules and proteins to specific mitochondrial subpopulations [75]. By combining mitochondrial function-enhancing drugs with targeted delivery technologies, it is possible to more effectively treat complex mitochondrial diseases while minimizing adverse effects.

**Figure 5:**
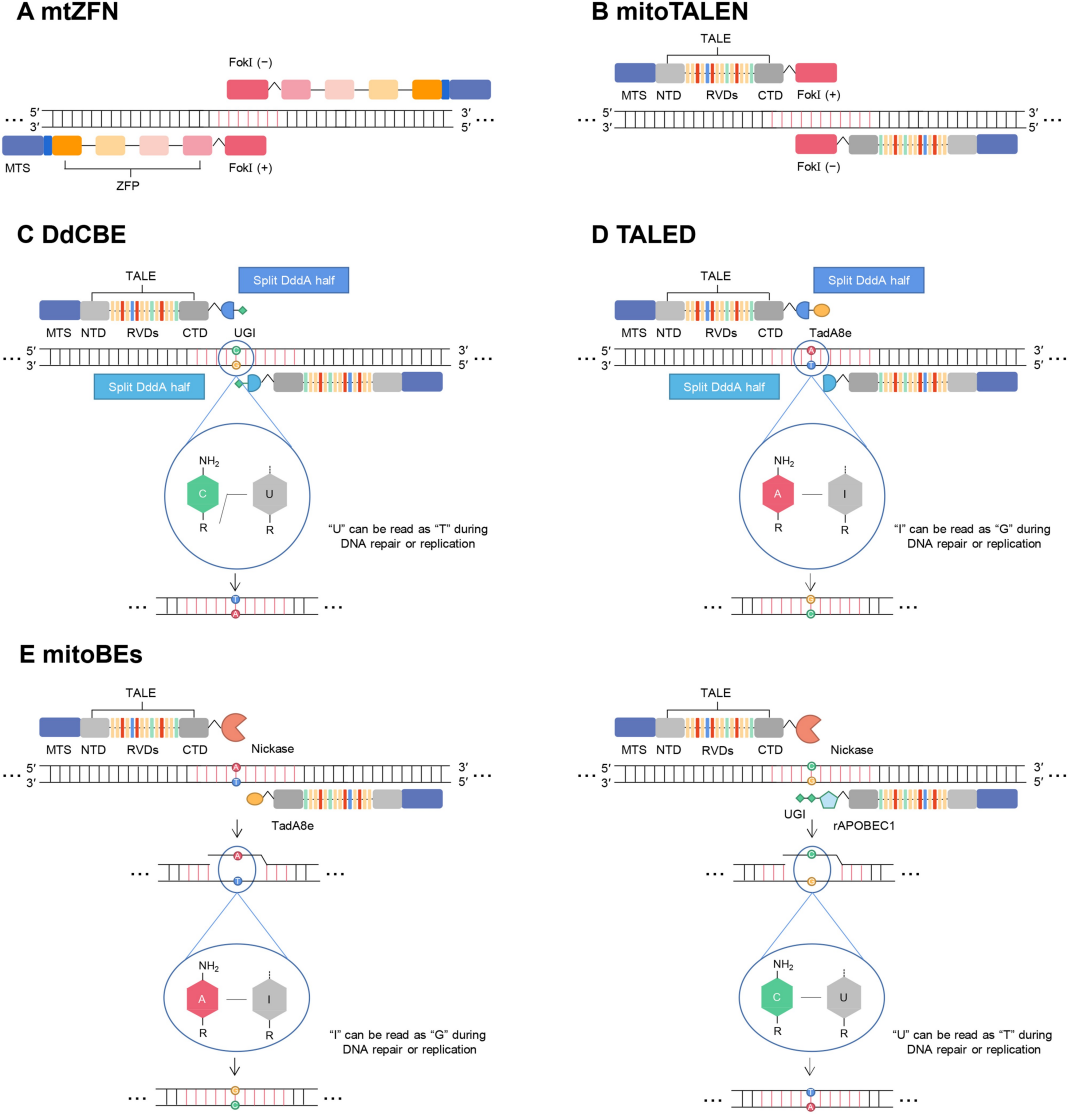
mtDNA editing techniques **A**. The mtZFN architecture combines two FokI domains with 3–4 ZFPs (the DNA recognition domain). **B**. The architecture of a dimeric mitoTALEN containing RVD sequences for DNA targeting and FokI nuclease domains. **C**. The DdCBE consists of a pair of MTS-TALE arrays, each linked to one half of DddA (from either the G1333 or G1397 split), and a UGI. The system catalyzes C-to-T transitions. **D**. The TALED system consists of DddA and TadA8e and catalyzes A-to-G transitions. **E.** mitoBEs integrate a nickase and a deaminase to achieve A-to-G or C-to-T base editing, depending on the type of deaminase used. mtZFN, mitochondrially targeted zinc finger nuclease; TALE, transcription activator-like effector; mitoTALEN, mitochondrially targeted TALE nuclease; DdCBE, DddA-derived cytosine base editor; DddA, double-stranded DNA deaminase toxin A; CTD, C-terminal domain; MTS, mitochondrial targeting sequence; NTD, N-terminal domain; UGI, uracil glycosylase inhibitor; ZFP, zinc-finger peptide; RVD, repeat-variable diresidue; TALED, transcription activator-like effector deaminase.

The primary approach for treating diseases caused by gene mutations is to eliminate the mutations in either nuclear DNA or mtDNA. The CRISPR system was reported to be unable to enter the mitochondrial matrix. Electron transport in mitochondria pumps protons out of the mitochondrial matrix, leaving it with a strong negative charge that prevents single-guide RNA (sgRNA) carrying the same negative charge [76]. As illustrated in **Figure 5**, several mtDNA editing tools have been developed, including protein-based mitochondrially targeted zinc finger nucleases (mtZFNs) [77] and mitoTALENs [78]; DdCBE [79], DdCBE variants [53], and a combined tool composed of other incision enzymes [80]; and mtDNA base editors (mitoBEs) [81]. These tools can cleave mutant mtDNA to reduce heteroplasmy while enabling single-base editing without disrupting the circular mtDNA structure. Given the multi-copy nature of mtDNA within cells, genotypic mosaicism and off-target effects are two significant challenges that must be addressed prior to the clinical application of gene-editing tools [24,80–83]. Single-cell mitochondrial genome sequencing technology offers considerable promise in tackling these challenges. It facilitates the detection of mitochondrial genomes in individual cells, enabling a comprehensive assessment of editing efficiency and off-target rates.

In summary, we believe that significant breakthroughs in single-cell mitochondrial research will occur over the next decade. For instance, the single-cell proteomics and metabolomics can be further combined to assess mitochondrial functions. Researchers will have a better understanding of pathogenesis, track the mitochondrial status dynamically, evaluate the therapeutic effect in mitochondrial disorders efficiently, and sequence mtDNA more cost-effectively. Furthermore, multi-omics data of individual mitochondria can also be integrated more easily and efficiently. These advances are poised to usher in a transformative era for early disease detection, precision prevention strategies, and personalized therapeutic interventions targeting mitochondrial dysfunction.

## CRediT author statement

**Xu Zhang:** Investigation, Writing – original draft, Visualization. **Peng An:** Investigation, Writing – original draft. **Zhengyang Zhang:** Writing – original draft. **Yanling Hao:** Supervision, Project administration. **Xiaoxian Guo:** Writing – original draft. **Yinhua Zhu:** Writing – review & editing. **Zhenglong Gu:** Conceptualization, Writing – review & editing. **Yongting Luo:** Conceptualization, Writing – review & editing, Funding acquisition. **Junjie Luo:** Conceptualization, Writing – review & editing, Supervision. All authors have read and approved the final manuscript.
